# Engineering of nicotianamine synthesis enhances cadmium mobility in plants and results in higher seed cadmium concentrations

**DOI:** 10.1111/tpj.70181

**Published:** 2025-04-29

**Authors:** Fabian Hollmann, Michael Weber, Mark G. M. Aarts, Stephan Clemens

**Affiliations:** ^1^ Plant Physiology University of Bayreuth D‐95440 Bayreuth Germany; ^2^ Laboratory of Genetics Wageningen University & Research 6700AA Wageningen Netherlands

**Keywords:** biofortification, food safety, micronutrient deficiency, metal homeostasis, metal chelators, nicotianamine, Cd accumulation, Cd contamination, *Arabidopsis thaliana*

## Abstract

Efficient biofortification, i.e., the enrichment of edible plant organs with micronutrients available for human consumption, is pursued through breeding and genetic engineering approaches. Enriching for iron (Fe) and zinc (Zn), two of the most critical trace elements, in cereal grains can be achieved by boosting the synthesis of nicotianamine (NA), a key metal chelator in plants. However, metal transport and distribution pathways are not entirely specific and may lead to the adventitious accumulation of potentially highly toxic non‐essential metals such as cadmium (Cd). We found evidence for the formation of intracellular Cd‐NA complexes driving Cd uptake and accumulation in two different yeast species and therefore studied *Arabidopsis thaliana* mutants as well as NA synthase overexpression lines in wild‐type and mutant backgrounds that showed varying degrees of NA deficiency or overproduction relative to controls. NA synthesis was enhanced by metal excess and conferred Cd and Zn tolerance. Importantly, when cultivated on soil containing environmentally relevant Cd levels, NA‐overproducing lines accumulated not only more Fe and Zn in their seeds but also more Cd. Thus, the engineering of NA synthesis can result in an unintended food safety risk that should be mitigated by carefully monitoring Cd phytoavailability in soils and, ideally, the use of low Cd germplasm for the engineering of biofortified crops.

## Significance Statement

Overproduction of the metal chelator nicotianamine is a widely applied approach to engineer biofortified crops with elevated Zn and Fe levels in their edible organs. Our study shows that this entails the risk of adventitiously enhancing Cd accumulation as well because nicotianamine drives Cd transport and mobility.

## INTRODUCTION

The mineral composition of plant seeds can affect human health in multiple ways. On the one hand, seeds provide a significant fraction of the dietary needs for essential mineral nutrients such as the microelements Fe and Zn (Gregory et al., 2017). On the other hand, seed consumption represents a major pathway of non‐occupational human exposure to potentially highly toxic environmental contaminants, most importantly arsenic (As) and cadmium (Cd) (Clemens & Ma, 2016; Zhao, Mcgrath, & Meharg, 2010). Both elements are nearly ubiquitously present in agricultural soils and can adventitiously accumulate in seeds or other edible plant organs.

Not all human populations have access to sufficient micronutrient quantities. Both Fe and Zn deficiency are highly prevalent. Globally, it is estimated that at least 2 billion people are affected by the suboptimal supply of micronutrients, most importantly Fe, Zn, vitamin A, and folate (Bailey, West Jr., & Black, 2015; Van Der Straeten et al., 2020). Current studies suggest that the number of people threatened by this so‐called ‘hidden hunger’ might be even higher. A recent re‐analysis of available population‐wide data on biomarkers for micronutrient status arrived at the conclusion that globally about 56% of preschool‐aged children and about 69% of non‐pregnant women of reproductive age are deficient in at least one of the most critical micronutrients (Stevens et al., 2022). A major symptom of insufficient Fe supply is anemia. Zn deficiency causes stunting in children and weakens the immune system. When quantified as Disability‐Adjusted Life Years (DALYs), the morbidity and mortality caused by micronutrient deficiencies are almost at the level attributable to chronic hunger, i.e., an undersupply of calories (Gödecke, Stein, & Qaim, 2018).

Human exposure to Cd and As is largely attributable to the intake of plant‐derived food containing trace amounts of these phytoavailable and potentially highly toxic elements. Arsenic is prone to accumulating in rice grown under flooded conditions. Both elements enter plant cells via transporters for essential or beneficial elements. For example, the dominant As species under reducing conditions, arsenite, is transported by aquaglyceroporins (Ma et al., 2008). Grains and vegetables account for most of the Cd intake in non‐smokers (Clemens, Aarts, Thomine, & Verbruggen, 2013; McLaughlin, Smolders, Zhao, Grant, & Montalvo, 2021). Cd uptake has been demonstrated to be mediated by members of the ZIP and Nramp families in particular (Zhao, Tang, Song, Huang, & Wang, 2022), thus via transporters involved in the transport of Fe, Zn, and other micronutrients. This makes Cd particularly problematic in the context of biofortification of crops for enhanced Fe, Zn, or other essential micronutrients if Cd is available to biofortified crops. Importantly, Cd is efficiently translocated from roots to above‐ground organs because it can be loaded into the xylem and phloem (Clemens & Ma, 2016; Zhao et al., 2022). Such *in planta* mobility underlies the potentially health‐threatening accumulation in seeds.

In recent years, the optimization of seed mineral composition, i.e., enriching for Fe and Zn, while ideally at the same time strongly reducing the accumulation of Cd (Van Der Straeten et al., 2020), has become a major breeding target. Substantial progress has been made regarding the former, most notably under the umbrella of the Harvest Plus project (Bouis & Saltzman, 2017; Li, Martin, & Fernie, 2024). More than 300 biofortified varieties of rice, wheat, maize, cassava, sweet potato, bean, and pearl millet have been developed with elevated levels of iron, zinc, or provitamin A in edible organs. More than 20 million people in low‐income countries already benefit from biofortification as a cost‐effective, sustainable strategy (www.harvestplus.org; Fao, 2019). For example, wheat varieties with 20–40% higher grain Zn are being cultivated in India and Pakistan (Velu et al., 2018). However, there are major limitations severely hampering further improvements. Zn and Fe accumulation in seeds are genetically complex traits, and natural variation, especially of grain Fe, is rather small (Vasconcelos, Gruissem, & Bhullar, 2017). Also, for practical reasons, breeding efforts have been largely restricted to only one micronutrient per crop, and it is not feasible to biofortify large numbers of established, locally adapted, high‐yielding varieties simultaneously (Van Der Straeten et al., 2020). Furthermore, it would be highly desirable to not only increase the concentrations of Zn and Fe in grains, but to enhance their bioavailability upon human consumption as well. Only a small fraction (about 5 to 20%, depending on food source) of the Fe and Zn in plant‐derived food is taken up into human gut cells (Hurrell, 2003). This is largely due to the presence of antinutrients such as phytate in seeds, which tightly binds Fe and Zn. A lowering of antinutrient concentrations or, better yet, higher seed concentrations of metal ligands that enhance bioavailability would therefore be highly desirable (Clemens, 2014).

Genetic interventions such as transgenesis, cisgenesis, or genome editing have the potential to overcome these limitations and make biofortification of crops far more efficient (Van Der Straeten et al., 2020). Attempts to engineer Fe and/or Zn content have mostly targeted three key steps in plant metal homeostasis, namely the uptake into cells, the *in planta* mobility, and the storage capacity. Typically used genes encode metal transporters of the ZIP family, the Fe storage protein ferritin, or enzymes involved in the synthesis of the metal chelator nicotianamine (NA) (Bhullar & Gruissem, 2013; Connorton & Balk, 2019). Increasing concentrations of NA in particular has been the most widely tested approach. Numerous studies have reported on plants overexpressing nicotianamine synthases (NAS). Pioneering studies with activation‐tagged rice lines showed that an overexpression of *OsNAS3* can result in much higher NA in seeds and more than twofold increases in seed Fe and Zn (Lee et al., 2009). Importantly, feeding studies with anemic mice showed that the transgenic seeds could rectify the causal Fe deficiency, whereas wild‐type seeds could not. This was explained by the larger fraction of seed Fe bound to a low molecular weight ligand other than phytate. Later, an even stronger effect on seed Zn was reported for lines with activation‐tagged *OsNAS2* (Lee et al., 2011). This time, mice fed the seeds recovered more rapidly from Zn deficiency than mice fed the wild‐type seeds. Thus, NA can enhance micronutrient concentrations in seeds as well as micronutrient bioavailability. Several studies have since then reported similar increases in seed Fe and/or seed Zn when an NAS is overexpressed either alone or in combination with a metal transporter and ferritin in rice (Wu, Gruissem, & Bhullar, 2019) or wheat endosperm (Beasley et al., 2019; Harrington et al., 2023). The hypothesized positive effects of NA overproduction on Fe and Zn bioavailability for human nutrition are supported by experiments with the Caco‐2 cell system (Beasley et al., 2020). Field trials showed that NAS overexpression can yield wheat seeds with elevated Fe and Zn levels and white flour with higher Fe bioavailability (Beasley et al., 2022), further demonstrating the biofortification potential of NAS.

The engineering of NA synthesis can have these effects because NA is a major metal chelator in plants and in monocots also a precursor of the phytosiderophore 2'‐deoxymugineic acid. NA can form stable complexes with Fe^2+^, Zn^2+^, and other divalent metal cations. Such complexation promotes the movement of metals within plants (Curie et al., 2009). In xylem sap, NA is the major ligand for Zn^2+^, Cu^2+^, Co^2+^, and Ni^2+^ (Flis et al., 2016). In the metal hyperaccumulator *Arabidopsis halleri*, constitutively elevated NA concentrations in the root are critical for the root‐to‐shoot translocation of Zn (Cornu et al., 2015; Deinlein et al., 2012). Transgenic *A. thaliana* plants overexpressing the tonoplast‐localized NA transporter ZIF1 retain more Zn in roots (Haydon et al., 2012). The same applies to *A. thaliana* mutants deficient in NA synthesis (Persson et al., 2016). In shoots, such mutants show a striking intercostal chlorosis phenotype in young leaves (Schuler, Rellán‐Álvarez, Fink‐Straube, Abadía, & Bauer, 2012), which was originally described for the tomato mutant chloronerva (Ling, Koch, Bäumlein, & Ganal, 1999). The localized chlorosis is due to Fe deficiency. NA is required for the phloem transport of Fe and Zn from source to sink leaves and to other phloem‐fed tissues (Schuler et al., 2012). This role in phloem transport is presumably a main reason why NA overproduction can result in higher Fe and Zn content of seeds. Also, NA may provide an additional metal‐binding sink in seeds.

The range of phenotypes reported for plants with altered NA synthesis indicates that metal binding by NA is not very specific. Generally, the pathways of elements within plants are intertwined, as they at least partially share ligands and transporters (Krämer, 2024). An important question for biofortification and food safety is therefore whether NA may also enhance the movement of potentially highly toxic elements such as Cd into seeds and other edible organs. Previous investigations had yielded two findings strongly suggesting a possible contribution of NA (synthesis) to Cd mobility. First, during the characterization of *A. thaliana* nicotianamine synthases in *Schizosaccharomyces pombe* cells as a heterologous system, we had observed that NA formation can weaken Cd tolerance, indicating a possible interaction of NA with Cd^II^ (Trampczynska, Kupper, Meyer‐Klaucke, Schmidt, & Clemens, 2010). Second, suppression of a nicotianamine synthase via RNA interference in the Zn and Cd hyperaccumulator *A. halleri* not only reduced the leaf accumulation of Zn but also the concentration of Cd in leaves (Uraguchi, Weber, & Clemens, 2019). We therefore initiated a study to investigate the effects of NA synthesis and especially of NAS overexpression on Cd tolerance and *in planta* mobility. Our data provide evidence for NA‐driven Cd accumulation in yeast cells and plants. Loss of NA synthesis in *A. thaliana* causes Cd and Zn hypersensitivity. Furthermore, we show that NAS overexpression can indeed mobilize Cd into seeds, demonstrating a potential food safety risk of respective biofortification approaches that needs to be addressed.

## RESULTS

### NA synthesis causes Cd hypersensitivity in *S. pombe* and *S. cerevisiae*

**Figure 1:**
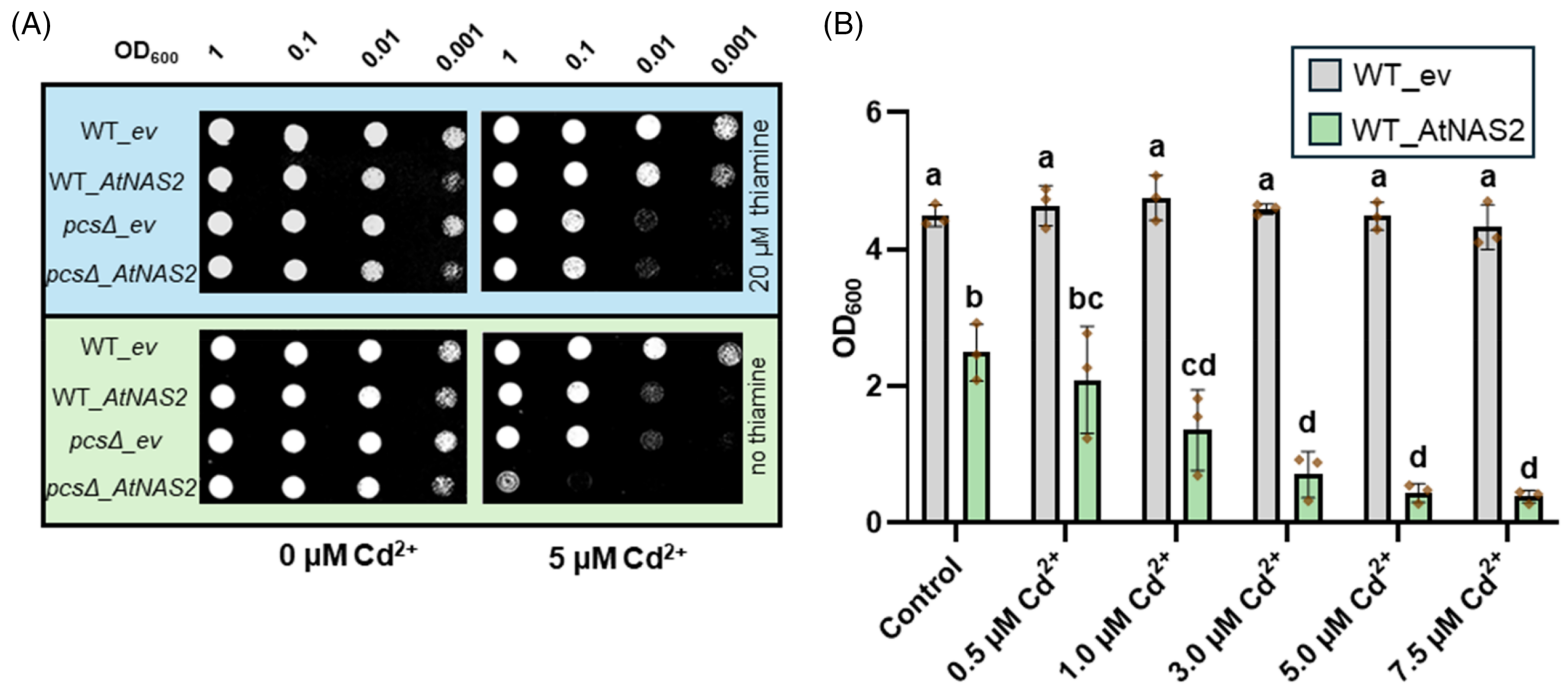
NA synthesis causes Cd hypersensitivity in *S. pombe*. (A) Suspensions of wild‐type and *pcs*Δ cells, carrying the empty vector (ev) or expressing AtNAS2, were diluted to ODs of 1, 0.1, 0.01, or 0.001 and dropped onto EMM plates with (control) or without 20 μm thiamine (which suppresses *AtNAS2* transcription) and with or without 5 μm Cd^2+^. Photos were taken after 48 h of incubation. (B) Wild‐type cells carrying the empty vector (ev) or expressing AtNAS2 were in addition tested in liquid culture (without thiamine). OD was determined after 20 h of growth in the presence of different Cd^2+^ concentrations. Shown are mean values ± SD (*n* = 3 independent experiments). Letters indicate significant differences according to two‐way anova and Tukey's post‐hoc test.

Expression of plant nicotianamine synthases in *S. pombe*, which naturally does not produce NA, and the corresponding NA synthesis enhance Zn, Ni, and Co tolerance while at the same time causing Cd hypersensitivity (Trampczynska et al., 2010). In order to investigate this effect further, we expressed AtNAS2 with an N‐terminal GFP tag in wild‐type and *pcs*Δ cells, the latter lacking the ability to detoxify Cd via the synthesis of phytochelatins (Clemens, Kim, Neumann, & Schroeder, 1999). All cells transformed with the GFP‐AtNAS2 construct showed a cytosolic GFP signal and accumulated up to about 5000 μg/g d.w. NA, while cells expressing C‐terminally tagged AtNAS2 accumulated about 20‐fold less NA. There were no significant differences in NA content between the genotypes expressing GFP‐AtNAS2. NA synthesis renders wild‐type cells Cd hypersensitive (Figure 1A). In liquid culture, strong NAS expression inhibits growth, yet NA‐dependent loss of Cd tolerance is still apparent (Figure 1B). Even the Cd hypersensitivity of *pcs*Δ is exacerbated. A comparable effect was observed in the other yeast model system, *Saccharomyces cerevisiae*. Both wild‐type and *ycf1* mutant cells, lacking a functional ABC type transporter for bis(glutathionato)Cd (Li et al., 1997), displayed higher Cd sensitivity when expressing AtNAS2 (Figure S1).

### NA‐dependent Cd hypersensitivity in *S. pombe* can be suppressed by an excess of competing metals

**Figure 2:**
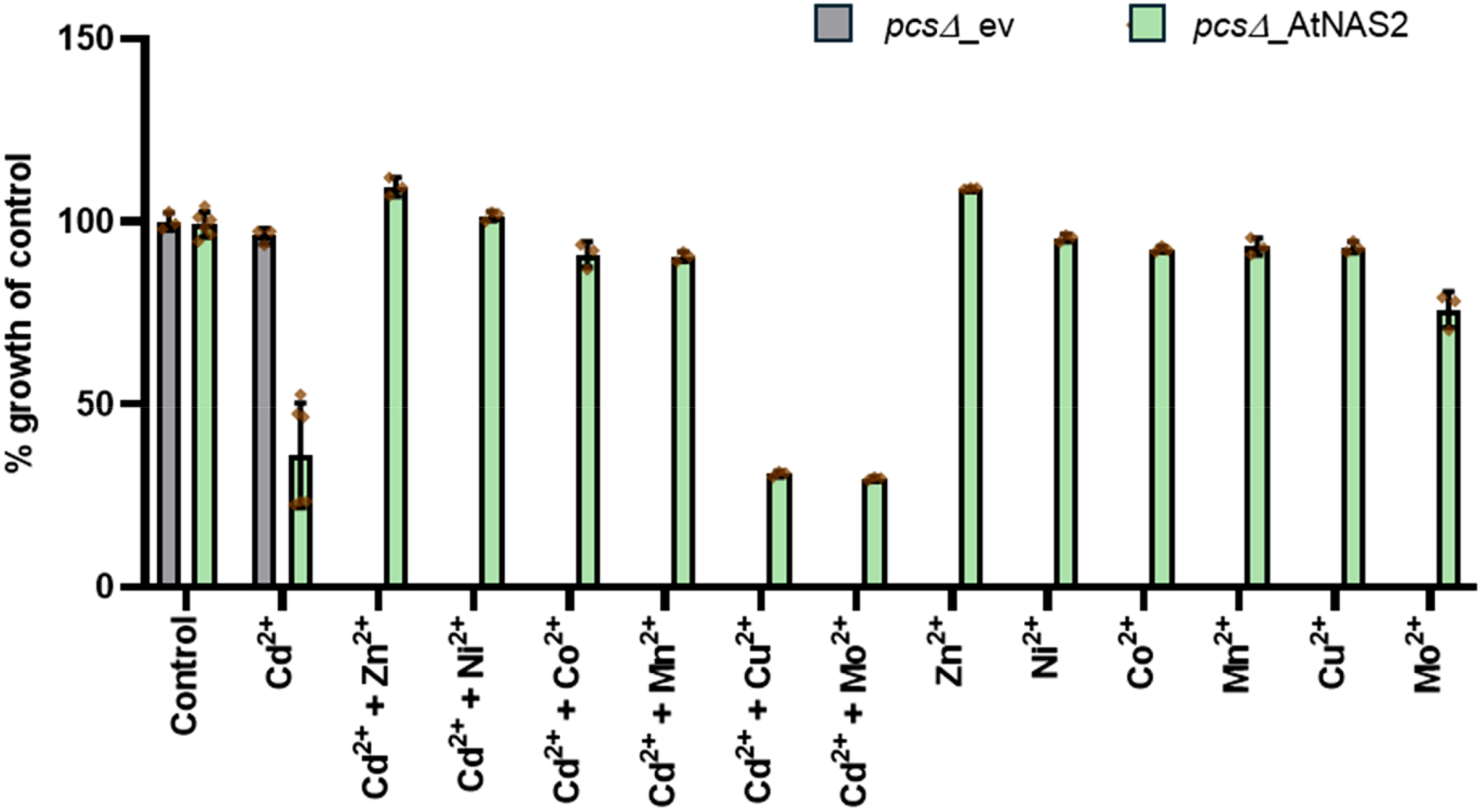
NA‐dependent Cd hypersensitivity in *S. pombe* is suppressed by cations able to form intracellular complexes with NA. Growth of *pcs*Δ cells expressing AtNAS2 was monitored in the presence of 0.3 μm Cd^2+^ either alone or plus Zn^2+^ (300 μm), Ni^2+^ (75 μm), Co^2+^ (5 μm), Cu^2+^ (0.3 μm), Mn^2+^ (150 μm) or Mo^2+^ (10 μm) relative to control conditions in the absence of any added cations. Concentrations of the added cations were chosen based on relative toxicity. Their growth effects are shown as well. For comparison, data for cells carrying the empty vector (ev) in the absence and presence of Cd^2+^ are indicated by the green bars. Shown are mean values ± SD (*n* = 3 independent experiments).

**Figure 3:**
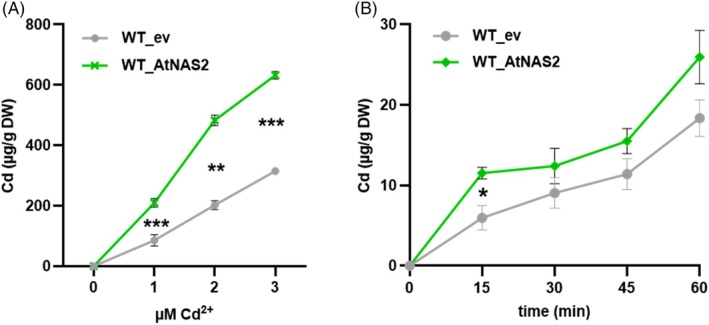
NA synthesis enhances Cd accumulation and uptake in *S. pombe*. (A) Wild‐type cells carrying the empty vector (ev) or expressing AtNAS2 were exposed to different Cd^2+^ concentrations. Cd content of cells was determined after 17 h of growth. (B) 6 h after transfer to thiamine‐free medium to activate AtNAS2 expression, cells were exposed to 12 μm Cd^2+^ and uptake was monitored for 60 min at 30°C and 4°C. Aliquots were harvested at the indicated time points, washed, and subjected to elemental analysis by ICP‐OES. Cd content of cells at 4°C indicated protein‐independent adsorption and was subtracted from the 30°C values. Shown are means ± SD (*n* = 3 independent experiments). Data for each Cd^2+^ concentration were analyzed by t‐test, *P < 0.05; ***P < 0.001.

When different micronutrient cations were added to *S. pombe pcs*Δ cells at concentrations below the toxicity threshold, we found partial suppression of NAS‐dependent Cd hypersensitivity by other cations known to form intracellular complexes with NA, namely Zn^2+^, Co^2+^, Mn^2+^ and Ni^2+^, while Cu^2+^ and Mo^2+^ had no such effect (Figure 2). This indicated the formation of intracellular NA‐Cd^II^ complexes and their suppression by an excess of outcompeting metals. We then analyzed whether NA synthesis influences Cd accumulation. When assayed after 17 h, i.e., at the beginning of the stationary phase, NAS‐expressing cells showed about a doubling of Cd levels relative to empty vector controls at all concentrations tested (Figure 3A). Also, they contained about twice as much Zn and about 3‐fold more Fe than controls (Figure S2). Mn accumulation was unaffected. Moreover, in short‐term uptake experiments performed 6 h after transfer to a thiamine‐free medium to induce AtNAS2 expression, cells took up more Cd than controls (Figure 3B). Thus, binding of Cd by NA may drive uptake of Cd into *S. pombe* cells. Alternatively, AtNAS2 expression may cause an apparent Zn deficiency, which could result in the up‐regulation of an uptake system adventitiously transporting Cd. However, no such transcriptional response was observed for the two Zn uptake candidate genes *zrt1* and *fet4* (Boch et al., 2008; Dainty, Kennedy, Watt, Bahler, & Whitehall, 2008). Transcript abundance was barely affected by AtNAS2 expression (Figure S3).

### NA‐deficient *A. thaliana* seedlings are Cd and Zn hypersensitive

**Figure 4:**
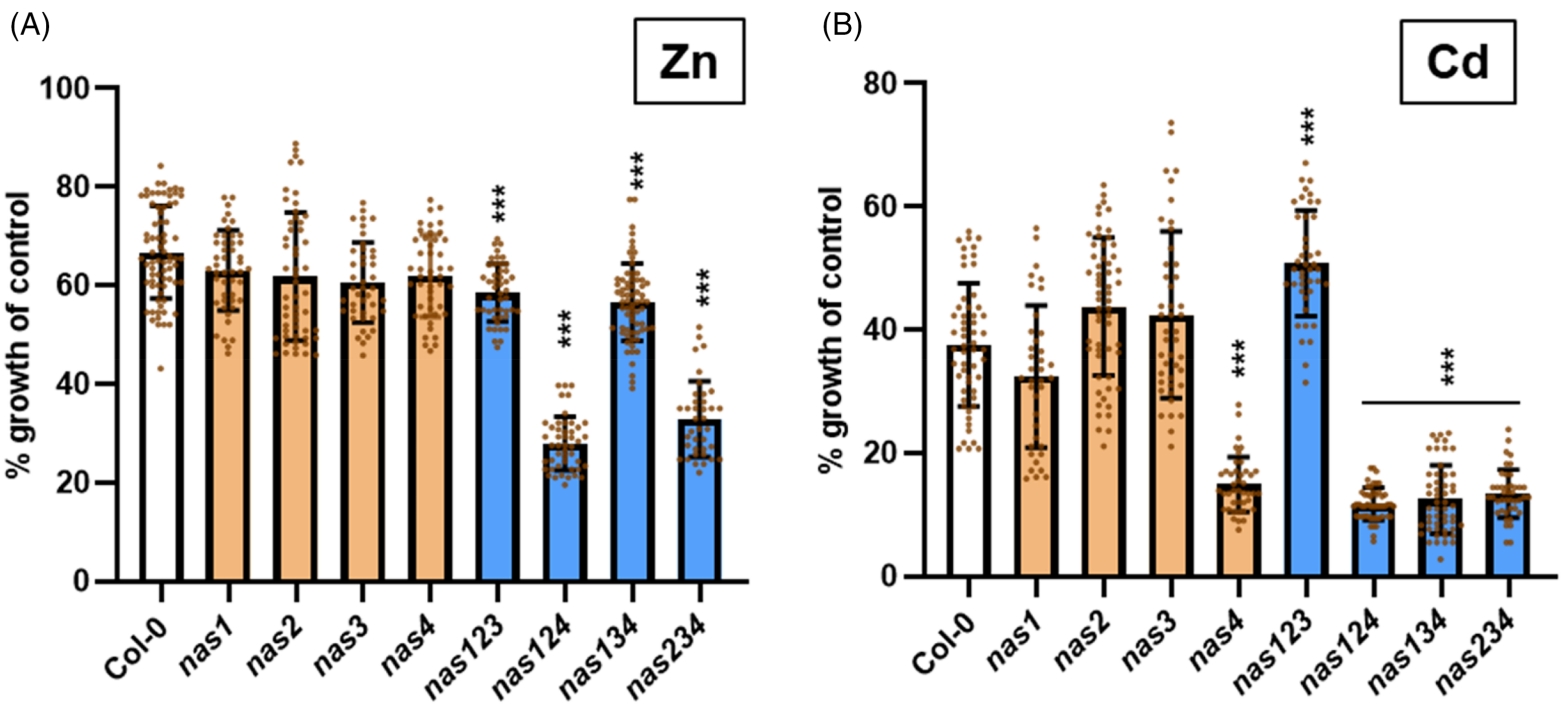
NA synthesis confers Cd and Zn tolerance to *A. thaliana*. Col‐0 and various *nas* single and triple mutant seedlings were grown on vertical plates in control medium or in the presence of either 80 μm Zn^2+^ (A) or 2.5 μm Cd^2+^ (B). Root length was measured after 12 days. Between 38 and 73 seedlings per genotype were analyzed in three independent experiments. Shown are means ± SD. Data were analyzed with the Kruskal–Wallis test followed by Bonferroni post‐hoc correction; ***P < 0.001.

The findings for NA‐synthesizing yeast cells prompted us to study a possible influence of NA on Cd‐related phenotypes in plants. We made use of a series of *A. thaliana nas* mutants carrying defects in up to three of the four *NAS* genes in the genome (Persson et al., 2016). The quadruple mutant was not included because it shows severe growth phenotypes already under control conditions (Schuler et al., 2012). Previous studies had demonstrated a contribution of NA synthesis to Ni tolerance (e.g., Douchkov, Gryczka, Stephan, Hell, & Baumlein, 2005; Klatte et al., 2009). We tested the Cd and, for comparison, Zn tolerance of seedlings grown vertically on agar plates. Reductions in root growth of Col‐0 caused by 2.5 μm Cd^2+^ or 80 μm Zn^2+^ were around 60% and 35%, respectively (Figure 4). Among the single mutants, only the *nas4* knockout line was significantly more compromised than Col‐0 when grown in the presence of Cd (Figure 4A). Among the triple mutants, all lines with a loss of NAS4 function showed Cd hypersensitivity comparable to the *nas4* mutant, while the *nas1nas2nas3* mutant grew like wild type. This indicated that only NAS4 contributes to NA‐dependent Cd detoxification. Under excess Zn conditions, none of the single mutants showed a stronger root growth reduction than Col‐0 (Figure 4B). Of the four tested triple mutants, *nas1nas2nas4* and *nas2nas3nas4* were clearly more Zn sensitive than Col‐0. The other mutants, *nas1nas2nas3* and *nas1nas3nas4*, were only slightly more affected than Col‐0. Thus, Zn detoxification appears to be mostly dependent on NAS2 and NAS4.

### *NAS* ‐overexpressing wild‐type and mutant plants accumulate more NA

**Figure 5:**
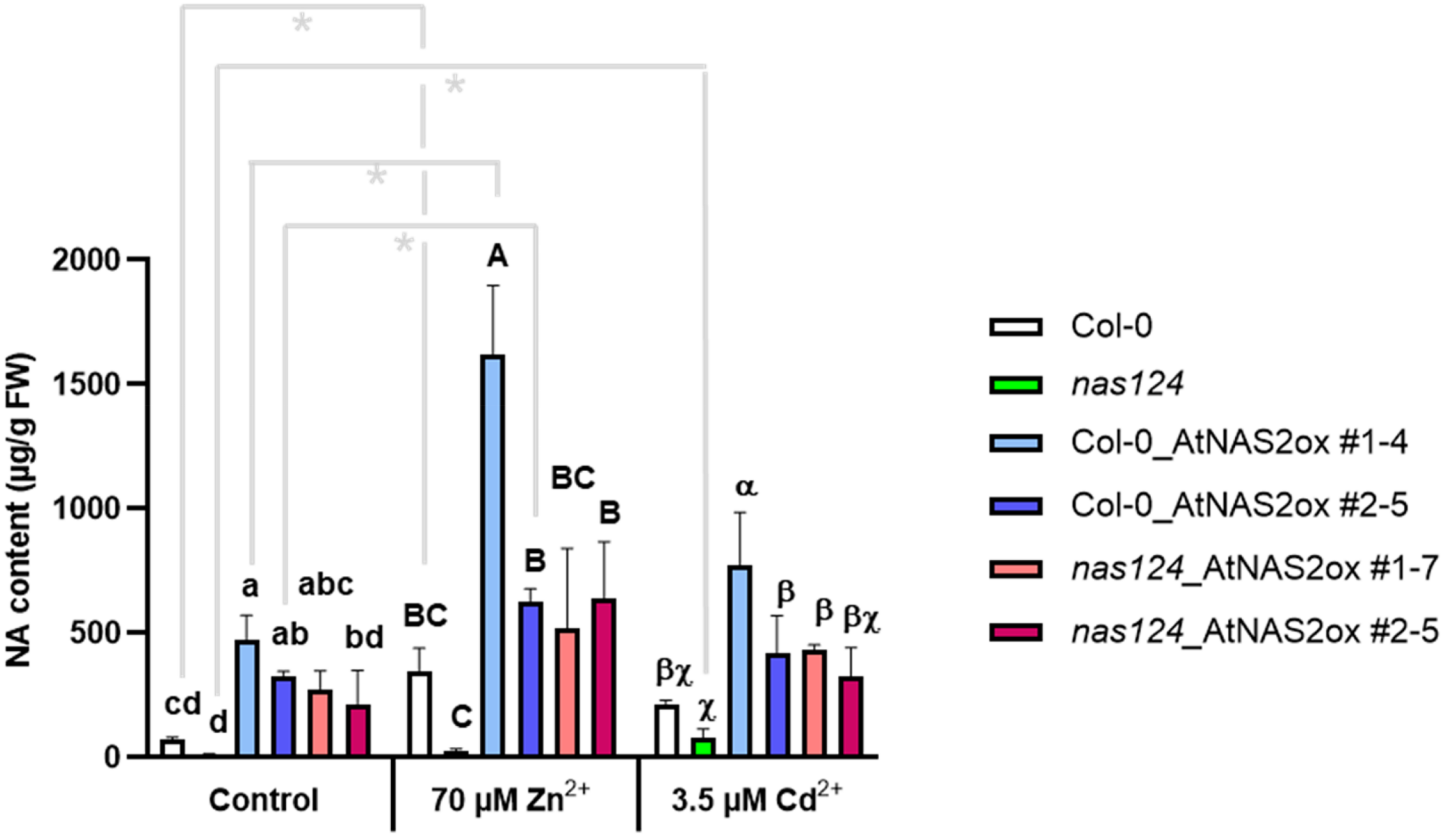
NA concentrations are altered in *nas* mutants and overexpression lines. Col‐0 and *nas1nas2nas4* seedlings (*nas124*) as well as two independent overexpression lines in the two backgrounds were grown on plates for 14 days in control medium or in the presence of either 70 μm Zn^2+^ or 3.5 μm Cd^2+^. NA concentrations in whole seedlings were measured by HPLC after Fmoc derivatization. Shown are means ± SD (*n* = 3; seedlings were pooled in each independent experiment). Letters indicate significant differences (*P* < 0.05) according to one‐way anova and Tukey's post‐hoc test. Asterisks denote significant differences in pairwise comparisons by t‐test, *P < 0.05.

The tolerance assays with *A. thaliana* seedlings had again indicated a possible interaction of NA with Cd. Next, we therefore addressed the question of whether NA influences the mobility and accumulation of Cd within plants. In order to cover a wide range of NA concentrations from deficiency to overproduction, we transformed Col‐0 and the most severely affected triple mutant *nas1nas2nas4* with *mCherry*‐*AtNAS2* under control of a strong constitutive promoter. Two homozygous lines each were selected for further studies. Expression of mCherry‐AtNAS2 rescued the *chloronerva*‐like phenotype of the *nas1nas2nas4* mutant (Figure S4), showing the functionality of the introduced transgene. Also, the leaf phenotype of the *nas1nas2nas4* mutant under conditions of Zn deficiency was complemented (Figure S4). Microscopic analysis revealed exclusively cytosolic localization of mCherry‐AtNAS2 (Figure S5). Quantification of NA in seedlings confirmed the NA deficiency of the *nas1nas2nas4* mutant and showed over‐accumulation of NA in the transgenic lines. Concentrations of NA were similar in the Col‐0 and triple mutant backgrounds and about 5‐8‐fold higher than in wild‐type Col‐0 (Figure 5). These results were confirmed for leaves of soil‐grown and hydroponically grown plants (Suppl. Figure S6). An analysis of NA concentrations in seedlings exposed to metal excess revealed that the presence of growth‐inhibiting Zn or Cd concentrations led to consistently higher NA accumulation (Figure 5). This applied to Col‐0 as well.

**Figure 6:**
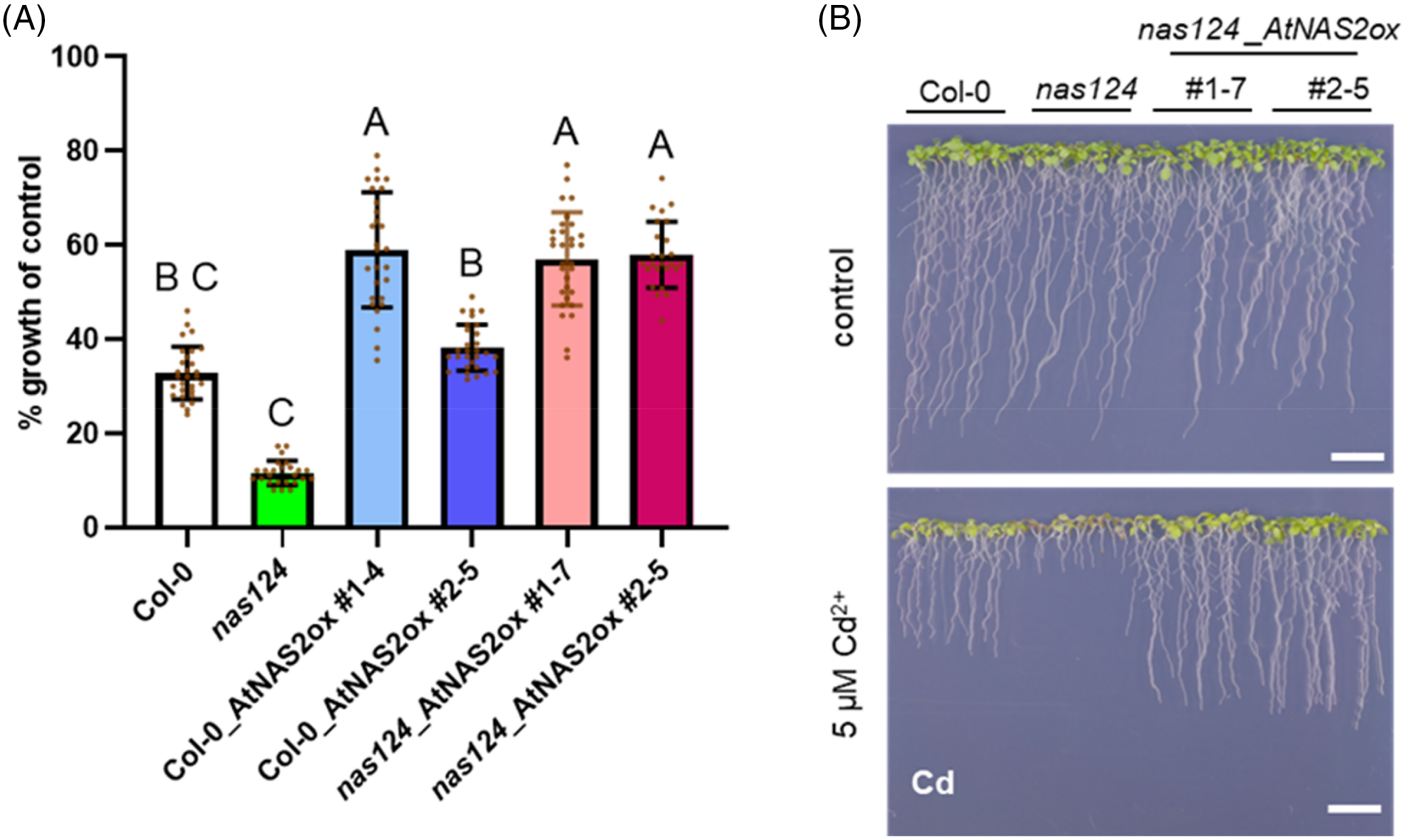
NA overproduction improves Cd tolerance in wild‐type and mutant background. Col‐0 and *nas1nas2nas4* seedlings as well as two independent overexpression lines in each of the two backgrounds were grown on vertical plates in control medium or in the presence of 5 μm Cd^2+^. (A) Root length was measured after 12 days. Between 25 and 31 seedlings per genotype were analyzed in three independent experiments. Shown are means ± SD. Data were analyzed with the Kruskal–Wallis test followed by Bonferroni post‐hoc correction. Letters indicate significant differences (*P* < 0.05). (B) Exemplary plate assays with Col‐0 and *nas1nas2nas4* seedlings as well as the two overexpression lines in the triple mutant background, scale bar = 1 cm.

Metal tolerance effects of NAS overexpression were again tested with vertically grown seedlings. The Zn and Ni hypersensitivity of the *nas1nas2nas4* mutant was fully complemented by mCherry‐AtNAS2. For Cd, an even higher tolerance than wild‐type level was observed for NAS overexpressors, regardless of genetic background (Figure 6).

### NA overexpression results in higher seed concentrations of Cd

Finally, we addressed the question most relevant for biofortification, namely the effects of NA overproduction on Cd accumulation in seeds. The design of the experiment aimed at exposing plants to environmentally relevant levels. Total Cd added to the artificially contaminated soil was 2 mg/kg, which is just 2fold higher than the threshold for risk assessment of agricultural soils (https://esdac.jrc.ec.europa.eu/themes/cadmium‐topsoils), a value that more than 5% of all soil samples in Europe exceed (Ballabio, Jones, & Panagos, 2024). Extraction with 0.1 N HCl yielded a Cd content of 1.73 ± 0.16 ppm, extraction with CaCl_2_ a Cd content of 0.026 ± 0.005 ppm (*n* = 5). The latter extraction method can be used as a proxy to assess phytoavailable Cd (Degryse, Broos, Smolders, & Merckx, 2003; Menzies, Donn, & Kopittke, 2007).

**Figure 7:**
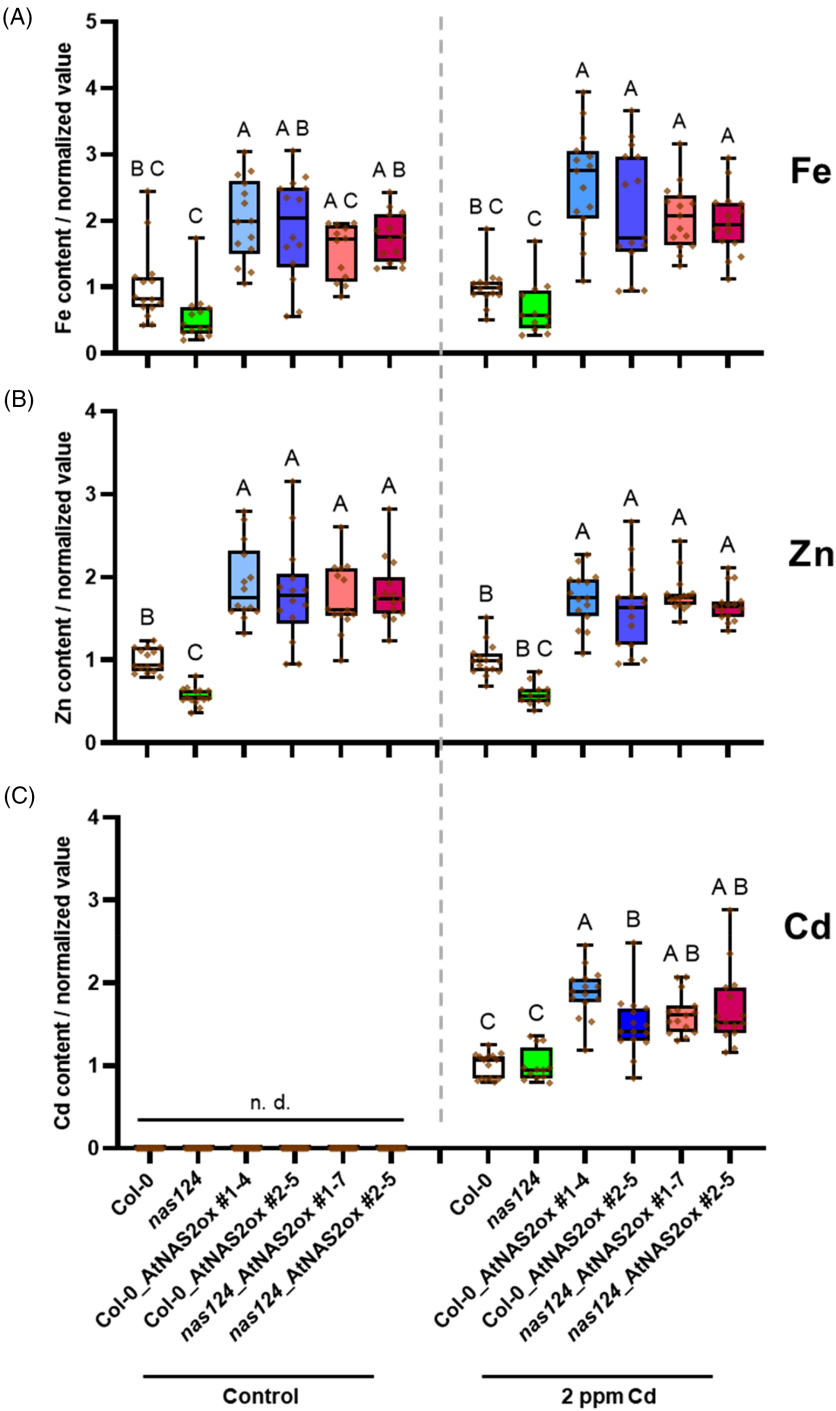
NA‐overproducing plants accumulate more Fe, Zn, and Cd in their seeds. Col‐0 and *nas1nas2nas4* plants (*nas124*) as well as two independent *NAS2* overexpression lines in the two genetic backgrounds were cultivated to maturity either on control soil or on soil artificially contaminated with Cd to an environmentally relevant extent. Ripened seeds were subjected to ICP‐OES analysis. Data were normalized to Col‐0 values. For each element, the mean for Col‐0 was set to 1 (control conditions for Fe and Zn, 2 ppm Cd conditions for Cd as no Cd was detectable under control conditions). Shown are means ± SD (10–15 individual plants cultivated in 3 independent experiments). Data were analyzed with the Kruskal–Wallis test followed by Bonferroni post‐hoc correction. Letters indicate significant differences (*P* < 0.05); n. d. = not detectable.

None of the genotypes displayed any toxicity symptoms during cultivation on Cd‐containing soil. Elemental profiling of seeds showed a significant reduction in Zn and Fe for the *nas1nas2nas4* triple mutant (Figure 7), both on control soil and on Cd soil. Manganese and the two macronutrients magnesium (Mg) and phosphorus (P) were not altered (Figure S7). Conversely, NA‐overproducing genotypes accumulated about 2–4‐fold more Fe and Zn than their respective parents on control soil. Background Cd contamination of the soil had only a minor effect on micronutrient content. Importantly, however, Cd accumulation in seeds was significantly elevated in all NA‐overproducing lines. In most cases, about a doubling was found to values around 10 μg/g (Figure 7, Figure S8).

## DISCUSSION

This study was motivated by the question of whether the engineering of crop plants for Zn and Fe biofortification may at the same time pose the food safety risk that unintended increases in the accumulation of non‐essential, potentially highly toxic elements occur. Specifically, we studied the effects of NA on Cd mobility within plants. Our data obtained through transfer or modulation of NA synthesis capacity in *S. pombe* and *A. thaliana*, respectively, support the hypothesis that NA can form intracellular complexes with Cd, increase Cd mobility within tissues, and ultimately enhance Cd accumulation even in seeds.

A key aspect of micronutrient accumulation in plant organs, besides the transport across membranes, is the mobility of the respective ions within cells and between cells. The reactivity of ions like Zn^2+^ requires tight control to suppress unfavorable interactions with biomolecules (Krämer, 2024). Only a minute fraction of intracellular Zn(II) is present in the ‘labile pool’, i.e., available for exchange, whereas most Zn(II) is bound to proteins (Clemens, 2022; Krężel & Maret, 2016). Binding partners in the labile pool are hypothesized to be mostly low molecular weight chelators such as NA (Krämer, 2024). However, the *in vivo* detection and identification of metal–ligand complexes remain notoriously difficult, because any extraction will likely destroy metal–ligand complexes and *in vivo* imaging techniques are not sensitive enough (Seregin & Kozhevnikova, 2023). Direct evidence for the formation of metal–ligand complexes in plants is thus largely restricted to easily accessible fluids such as the xylem (Flis et al., 2016) and the liquid endosperm of peas (Grillet et al., 2014). The functions of low molecular weight chelators therefore have to be inferred from *in vitro* data and the phenotypes caused by the modulation of chelator synthesis in model systems.

Pathways that deliver micronutrients to target sites including storage compartments are not perfectly specific and interfere with each other. This applies to low molecular weight chelators as well. When NA and other ligands form complexes with, for example, divalent cations, the stability of such complexes is, independent of the atoms involved in chelation, described by the Irving‐Williams series: Mn^II^ < Fe^II^ < Co^II^ < Ni^II^ < Cu^II^ > Zn^II^ (Irving & Williams, 1948). Reported *in vitro* data for NA complexes are in accordance with that (Benes, Schreiber, Ripperger, & Kircheiss, 1983; Curie et al., 2009). Cd^II^ was originally not studied. However, more recent data obtained through direct infusion electrospray ionization mass spectrometry provided *in vitro* evidence for stable Cd‐NA complexes (Rellan‐Alvarez, Abadia, & Alvarez‐Fernandez, 2008). Their formation was strongly favored by pH values above 6.5, which would suggest the existence of such complexes in the cytosol and the phloem.

Results of our *S. pombe* experiments are consistent with the formation of cytosolic Cd‐NA complexes. NA‐synthesizing cells showed enhanced accumulation not only of Zn and Fe (Figure S3) but also of Cd in a dose‐dependent manner (Figure 3A). This effect was even observed in short‐term Cd exposure experiments performed shortly after activation of AtNAS2 expression via transfer of cells into a thiamine‐free medium (Figure 3B). It is therefore unlikely that the effects of NA synthesis on the micronutrient status indirectly cause an up‐regulation of Cd uptake pathways. This is supported by the lack of up‐regulation of the two known Cd uptake pathways in *S. pombe*, Zrt1 or Fet4 (Boch et al., 2008; Dainty et al., 2008) (Figure S2). Rather, Cd‐NA complex formation acts as a driving force for uptake.

In *S. pombe* cells, the stronger Cd accumulation results in Cd hypersensitivity (Figure 1), an effect that was confirmed in *S. cerevisiae* cells (Figure S1). Interestingly, the NA effect on Cd tolerance can be suppressed by a non‐toxic excess of other metal cations known to form complexes with NA, namely Mn^2+^, Fe^2+^ and Zn^2+^, while Mo^2+^ excess had no such effect (Figure 2). The exception is Cu^2+^, which is the most tightly controlled metal in cells as it forms the most stable complexes regardless of the type of ligand involved, in accordance with the Irving‐Williams series (Irving & Williams, 1948). Unlike the other metals, it is therefore generally assumed that no labile Cu pool exists, i.e., that it is not bound by low molecular weight chelators but by designated metallochaperones instead (Krämer, 2024; Rae, Schmidt, Pufahl, Culotta, & O'Halloran, 1999). Overall, the competition experiments therefore support the hypothesized existence of cytosolic Cd‐NA complexes.

In plants, the chelation of Cd by NA could potentially enhance the root‐to‐shoot mobility of Cd. Ample evidence demonstrates key roles of NA in the long‐distance transport of metals within plants and in tolerating metal excess conditions (reviewed in Seregin & Kozhevnikova, 2023). NA was identified as the factor normalizing the striking intercostal chlorosis phenotype of the tomato mutant *chloronerva*. Initial work was therefore focused on Fe (Pich, Manteuffel, Hillmer, Scholz, & Schmidt, 2001). Detailed analysis of *A. thaliana nas* mutants showed a specific defect of NA‐deficient plants in unloading Fe from the phloem into sink tissues such as young leaves and reproductive organs (Schuler et al., 2012). This is different from the contribution of NA to Zn mobility. Studies with the metal hyperaccumulating species *A. halleri* and with *A. thaliana* demonstrated an involvement of NA in loading Zn into the root xylem. *A. halleri* lines with reduced root NA synthesis (Cornu et al., 2015) as well as NA‐deficient *A. thaliana* mutants (Persson et al., 2016) retain more Zn in the root. This explains the lower Zn accumulation in above‐ground organs when NA levels are insufficient. Furthermore, NA chelates Zn in the phloem as indicated by the reproductive defects in *A. thaliana nas* quadruple mutants, which can be rescued by the combined application of Fe and Zn (Schuler et al., 2012).

While the functions of NA in long‐distance transport of Fe and Zn depend on the formation of complexes with these metals in the cytosol and the phloem, metal tolerance effects of NA can be explained by cytosolic complex formation alone, i.e., an intracellular buffering function (Seregin & Kozhevnikova, 2023). Ectopic NAS expression was shown to confer pronounced Ni tolerance (e.g., Douchkov et al., 2005). Conversely, NA deficiency causes Ni hypersensitivity in *A. thaliana* (Klatte et al., 2009) and *A. halleri* (Cornu et al., 2015). Regarding a contribution of NA to Zn tolerance, previously published studies had produced conflicting results. No effect was observed for higher order *A. thaliana nas* mutants, whereas under different experimental conditions, a *nas4* mutant was found to be Zn hypersensitive (Palmer, Hindt, Schmidt, Clemens, & Guerinot, 2013). Our seedling assays clearly establish a significant role of NA in dealing with Zn excess. Specifically, *NAS2* and *NAS4* contribute to Zn detoxification as evident from the growth defect in triple mutants lacking these two genes (Figure 4). Equally evident is NA‐dependent Cd detoxification. Not only were some of the mutant lines Cd hypersensitive, NA overproduction enabled even the *nas1nas2nas4* triple mutant to grow better than Col‐0 in the presence of Cd (Figure 6). Interestingly, the loss of NAS4 function is sufficient to cause Cd hypersensitivity, as was shown previously (Koen et al., 2013). This may indicate the existence of distinct NA pools in *A. thaliana* roots (Palmer et al., 2013) and detoxification of Zn and Cd at different sites within the root.

The distinctive yet similarly positive effects of NA on the long‐distance transport of the two most important mineral micronutrients, Fe and Zn, motivated numerous studies employing the engineering of NAS expression in crop plants to alleviate respective micronutrient deficiencies. This applies to Fe in particular because agronomic strategies and conventional breeding have so far shown only low efficiency (Balk et al., 2019). Target crops have been predominantly rice and wheat. A recent compilation of publications describing attempts to engineer biofortified rice, for example, listed 33 publications describing transgenic approaches (Wairich, Ricachenevsky, & Lee, 2022). In 13 of them, an *NAS* gene was employed, illustrating the key importance of modulating NA concentrations. Initially, transgenic plants were assayed under lab and greenhouse conditions. In most studies, increases in grain Fe and/or Zn greater than twofold were achieved. Recently, for example, the combined overexpression of a vacuolar Fe transporter gene, *TaVIT2‐D*, and a rice *NAS* gene, *OsNAS2*, in wheat enhanced the Fe and Zn concentration in flour derived from the transgenic plants by three‐ and twofold, respectively. Importantly, in some projects field studies have already been performed with *NAS*‐overexpressing plants. They confirmed the beneficial effects on grain Fe and Zn (Beasley et al., 2022). After the transfer of selected *NAS* transgenic events into *indica* rice cultivars grown in Bangladesh and the Philippines, the application of *NAS* overexpression for alleviating hidden hunger may be close (Tsakirpaloglou et al., 2023).

Such promising endeavors are further supported by accumulating evidence demonstrating the second major advantage of *NAS* genes for biofortification, namely the positive effect on Fe and Zn bioavailability for humans. Pioneering studies with grains of NAS‐overexpressing rice lines had already suggested this based on feeding trials with mice (Lee et al., 2009, 2011). When the bioaccessibility of Fe and Zn in wheat flour containing elevated NA concentrations was analyzed through simulated digestion, much stronger release, especially of Fe, was detected relative to flour derived from WT plants (Harrington et al., 2023). Field‐grown transgenic wheat plants yielded flour with improved Fe bioavailability according to *in vitro* digestion and application to Caco‐2 cells (Beasley et al., 2022), confirming earlier results obtained in a chicken model (Beasley et al., 2020).

Given the proven potential of *NAS* engineering to achieve biofortification beyond what is possible by conventional breeding, it is essential to address the question of whether NA overproduction could have detrimental effects on food quality as well. Chronic exposure to Cd, which potentially accumulates in the human body for >30 years, represents a well‐recognized health risk associated with kidney damage, osteoporosis, and cancer (EFSA, 2009). Recent risk assessments found that the safety margin between exposure and toxic effects is small or non‐existent (Nordberg et al., 2018). Because Cd is readily taken up by plants and is mobile within plants, background accumulation in edible crop tissues accounts for most of the Cd exposure (Yu, Alseekh, Zhu, Zhou, & Fernie, 2024). Although Cd is a ubiquitously present contaminant in agricultural soils (McLaughlin et al., 2021), the possible mobilization of Cd to harvested plant parts has not been addressed in the vast majority of studies. An exception is a study on rice plants overexpressing *OsNAS1* and a barley NA aminotransferase gene, HvNAATb, involved in phytosiderophore synthesis (Banakar et al., 2017). Here, one of the transgenic lines showed reduced Cd accumulation in the unpolished grain after growth in hydroponic culture with 10 μm Cd added to the medium. All three tested lines contained less Cd in their endosperm. It was hypothesized that these findings are explained by competition of elevated Fe and Zn levels for seed loading and by a downregulation of the *OsLCT1* gene in the transgenic lines. The transporter OsLCT1 has been implicated in the mobilization of Cd towards the seeds in rice (Uraguchi et al., 2011; Uraguchi, Kamiya, Clemens, & Fujiwara, 2014).

In contrast, our results obtained for soil‐cultivated *A. thaliana* plants subjected to environmentally relevant Cd exposure conditions strongly suggest enhanced NA‐mediated mobilization of Cd into seeds (Figure 7), a finding consistent with the formation of Cd‐NA complexes in the cytosol and phloem discussed above. Several lines of evidence support the notion that the observations for the model *A. thaliana*, cultivated under controlled lab conditions, are transferable to crop plants. First, phenotypes of the transgenic lines expressing an *NAS* gene under the control of a strong constitutive promoter closely resembled those of *NAS*‐overexpressing rice and wheat lines. NA concentrations were elevated by a factor of 5–8, similar to reported values for rice and wheat (Harrington et al., 2023; Lee et al., 2009), and explained by the good correlation between *NAS* transcript abundance and NA concentrations in plant tissues (e.g., Deinlein et al., 2012; reviewed in Seregin & Kozhevnikova, 2023). In seeds, the constitutively elevated NA concentrations resulted in about twofold higher Fe and Zn concentrations, which again is equivalent to the effect size in *NAS‐overexpressing* rice and wheat (Beasley et al., 2019; Wu et al., 2019). Second, the Cd exposure strength was in a range that is relevant for Cd‐polluted agricultural soil. Total Cd of the soil (2 mg/kg) was about 6‐fold higher than the global average background soil Cd concentration of 0.36 mg/kg (Kubier, Wilkin, & Pichler, 2019) and only twice as much as the threshold considered as contamination (Ballabio et al., 2024). CaCl_2_ extractions, which are widely accepted as surrogate for the soil solution (McLaughlin et al., 2021), showed a concentration of ca. 26 μg/L, about five times the upper value for soil solution Cd released by weathering (Kubier et al., 2019) and in the range reported for Cd‐contaminated soils (Lorenz et al., 1997). Third, the Cd accumulation observed in seeds of *NAS*‐overexpressing *A. thaliana* plants (around 10 mg/kg) was only about 4–5 times higher than what has been found in, for example, rice grains harvested on Cd‐contaminated rice paddy soils (Chen, Wang, Chang, Kopittke, & Zhao, 2020). Thus, it appears highly likely that *NAS*‐overexpressing crop plants will accumulate more Cd in their seeds when cultivated on soil with moderate and possibly even background levels of Cd.

Since Cd is a class I carcinogen, there is no safe human exposure level, and the intake from plant‐derived food should be as low as reasonably achievable (Nordberg et al., 2018; Smolders & Mertens, 2013). Thus, the highly desirable use of biofortified crops with engineered NA synthesis or transport should be accompanied by careful monitoring of the soil where such lines are planted. Also, the field testing that precedes widespread planting should include cultivation on soils differing in phytoavailable Cd. Arguably, the most effective measure would be the combination of engineered biofortification with a low Cd trait. There is ample evidence available that at least in rice, a rather simple genetic intervention, the inactivation of *OsNramp5*, a gene encoding a Mn transporter, can drastically reduce the uptake of Cd (e.g., Ishikawa et al., 2012; Sasaki, Yamaji, Yokosho, & Ma, 2012).

## MATERIALS AND METHODS

### Yeast culture and heterologous expression

*Schizosaccharomyces pombe* cells were cultivated at 30° in either Edinburgh's minimal medium or in YE5S medium. Minimal and complex media used for *Saccharomyces cerevisiae* were YNB and YPD, respectively. GFP‐tagged AtNAS2 was expressed in the *S. pombe* mutant strains *Δpcs* (Clemens et al., 1999) and *ΔpcsΔzhf* (Clemens et al., 2002) using pSGP72. Thiamine was added to modulate expression strength from the *nmt1* promoter as indicated. Expression in *S. cerevisiae* was driven by the *GAL1* promoter in pYES2. Metal tolerance and metal accumulation were analyzed in liquid culture, with metal tolerance additionally analyzed in drop dilution assays.

### Plant cultivation and metal exposure

*Arabidopsis thaliana* wild type (Col‐0) and a series of single and triple *nas* mutants (Persson et al., 2016) were analyzed. Also, Col‐0 and the *nas1nas2nas4* triple mutant were transformed with *mCherry‐AtNAS2* under the control of the *UBQ10* promoter. Seeds were surface sterilized in chlorine gas and then stratified for 48 h at 4°C in the dark. Seedlings were cultivated under long‐day conditions (16 h light/8 h dark) at 22 ± 2.5°C on a modified 1/10 Hoagland medium: 0.28 mm Ca(NO_3_)_2_, 0.1 mm (NH_4_)H_2_PO_4_, 0.2 mm MgSO_4_, 0.6 mm KNO_3_, 5 μm of a complex of Fe(III) and *N*,*N*´‐di‐(2‐hydroxybenzoyl)‐ethylenediamine‐*N*,*N*´‐diacetate (HBED; ABCR GmbH, Karlsruhe, Germany), 5 mm MES, 1% (w/v) sucrose, pH 5.7, 1% (w/v) Type E agar (Sigma‐Aldrich, Taufkirchen, Germany). Different concentrations of Zn^2+^ or Cd^2+^ (as sulphate and chloride salts, respectively) were added as indicated.

For hydroponic culture in modified 1/10 Hoagland medium, seeds were placed in agar‐filled PCR tubes and cultivated in pipette‐tip boxes for 3 weeks before transfer into 50 ml Falcon tubes. After an additional three to 4 weeks under short‐day conditions (8 h light/16 h dark) at 22 ± 2.5°C, plants were treated as indicated. Medium was renewed weekly to ensure sufficient oxygen and mineral supply.

### Soil experiments

For experiments investigating Cd accumulation in seeds, soil was artificially contaminated with CdCl_2_ as follows: mineral soil was sieved (to remove organic material and stones) and dried at 60°C for 24–48 h. 100 ml of CdCl_2_ solution (89 μm) was added per 500 g of soil. The suspension was mixed for 3–4 h using a REAX 20 overhead shaker (Heidolph‐Instruments, Schwabach, Germany). Afterwards, soil was dried again, crushed, and mixed with perlite (2:1 [soil: perlite]). Soil Cd concentration was determined after three extractions (2 g of soil in 10 ml solution) with 0.1 N HCl as well as after three extractions with 10 mm CaCl_2_. Twenty‐four hours before seedling transfer, the soil/perlite mixture was moistened. Seedlings were pre‐cultivated on 1/10 modified Hoagland medium under short‐day conditions (8 h light [23°C], 16 h dark [18°C]) for about 14 days before transfer. After transfer, plants were grown under short‐day conditions (8 h light [23°C], 16 h dark [18°C]) for an additional 3 weeks. Afterwards, the plants were cultivated under long‐day conditions (16 h light [23°C], 8 h dark [18°C]) until seed maturation. Plants were placed randomly.

### Nicotianamine analysis

NA concentrations were determined after 9‐fluorenylmethyl chloroformate (Fmoc) derivatization by HPLC, essentially following a protocol established by Klatte et al. (2009). Lyophilized yeast cells or dried plant material were extracted in Millipore H_2_O at 80°C. Following centrifugation, 25 μL supernatant was transferred to a lightproof Eppendorf tube and mixed with 75 μL Na‐borate buffer (0.5 M boric acid, 50 mm Na‐EDTA, pH 7.7). The addition of 50 μL Fmoc (12 mm, dissolved in acetone) initiated derivatization for 45 sec. Excess Fmoc was precipitated with 50 μL 1‐adamantylamine hydrochloride (40 mm, in 3:1 acetone:water) and removed by centrifugation. 5 μL supernatant was injected on a Nucleosil 100‐5 C18 column (5 μm particle size) (Macherey‐Nagel, Düren, Germany), equilibrated with buffer A (20% acetonitrile in 50 mm sodium acetate, pH 4.2). For separation of nicotianamine, the following binary gradient was used (flow rate 1 ml/min): 0–5 min (0% buffer B, 20% acetonitrile in 50 mm sodium acetate, pH 4.2), 5–15 min (20% buffer B, linear), 15–20 min (100% buffer B, linear), 20–22 min (100% buffer B), 22–24 min (0% buffer B, linear), 24–34 min (0% B). The temperature of the column was 40°C. Fmoc‐derivatized compounds were detected with a fluorescence detector (FP‐2020_Plus_ Jasco, excitation wavelength: 263 nm; emission wavelength: 313 nm). Authentic NA standard (TRC, Toronto Research Chemicals, Vaughan, Canada) was used for quantification.

### Elemental analysis of yeast, plant and soil samples

In order to desorb cations, roots and yeast cells were washed at 4°C with Millipore water, then twice with 20 mm CaCl_2_, twice with 10 mm Na‐EDTA, pH 5.7, and finally again with Millipore water. Leaves were rinsed with Millipore water. Yeast cells were frozen and lyophilized; plant material was dried. After transfer into 2 ml 65% HNO_3_ and 1 ml H_2_O_2_, samples were digested using a START‐Microwave (MLS GmbH, Leutkirch, Germany). Diluted samples were analyzed with an ICP‐OES (ICAP‐6500 DUO; Thermo Fisher‐Scientific, Erlangen, Germany). Soil extracts were diluted and directly analyzed.

### Confocal microscopy

The subcellular localization of tagged AtNAS2 was determined by visualizing GFP or mCherry fluorescence with a SP5 confocal laser‐scanning microscope (Leica Microsystems, Wetzlar, Germany). Suspended *S. pombe* cells were mixed with low‐melting agarose (0.8%) prior to analysis. *A. thaliana* seedlings were analyzed after 7 days of cultivation on vertical plates.

### Statistical analysis

Statistical analysis was performed with R studio as indicated.

## Supporting information


**Figure S1.** NA synthesis causes Cd hypersensitivity in *S. cerevisiae*.
**Figure S2.** NA synthesis enhances Zn and Fe accumulation in *S. pombe*.
**Figure S3.** Transcript abundance of the two *S. pombe* Zn deficiency marker genes *zrt1* and *fet4* is not affected by AtNAS2 expression.
**Figure S4.** AtNAS2 overexpression rescues the *chloronerva* phenotype of the *nas1nas2nas4* triple mutant and confers wild‐type growth under Zn deficiency conditions.
**Figure S5.** Cytosolic localization of mCherry‐AtNAS2 expressed under control of the UBQ10 promoter.
**Figure S6.** Leaf NA concentrations in Col‐0, the *nas1nas2nas4* triple mutant, and overexpression lines.
**Figure S7.** Effects of NA overproduction on the accumulation of selected macro‐ and microelements as well as Cd in seeds.
**Figure S8.** Effects of NA overproduction on the accumulation of Fe, Zn and Cd in seeds.

## Data Availability

The data that support the findings of this study are available from the corresponding author upon reasonable request.
