# Supplementary material for: Engineering of nicotianamine synthesis enhances cadmium mobility in plants and results in higher seed cadmium concentrations
Source: Plant J. 2025 Apr 29;122(2):e70181. doi: 10.1111/tpj.70181 (PMC12040310; doi:10.1111/tpj.70181)
Supplement: Supplementary file 1 — Figure S1. NA synthesis causes Cd hypersensitivity in S. cerevisiae. Figure S2. NA synthesis enhances Zn and Fe accumulation in S. pombe. Figure S3. Transcript abundance of the two S. pombe Zn deficiency marker genes zrt1 and fet4 is not affected by AtNAS2 expression. Figure S4. AtNAS2 overexpression rescues the chloronerva phenotype of the nas1nas2nas4 triple mutant and confers wild‐type growth under Zn deficiency conditions. Figure S5. Cytosolic localization of mCherry‐AtNAS2 expressed under control of the UBQ10 promoter. Figure S6. Leaf NA concentrations in Col‐0, the nas1nas2nas4 triple mutant, and overexpression lines. Figure S7. Effects of NA overproduction on the accumulation of selected macro‐ and microelements as well as Cd in seeds. Figure S8. Effects of NA overproduction on the accumulation of Fe, Zn and Cd in seeds. [file TPJ-122-0-s001.pdf]

Suppl. Fig. S1

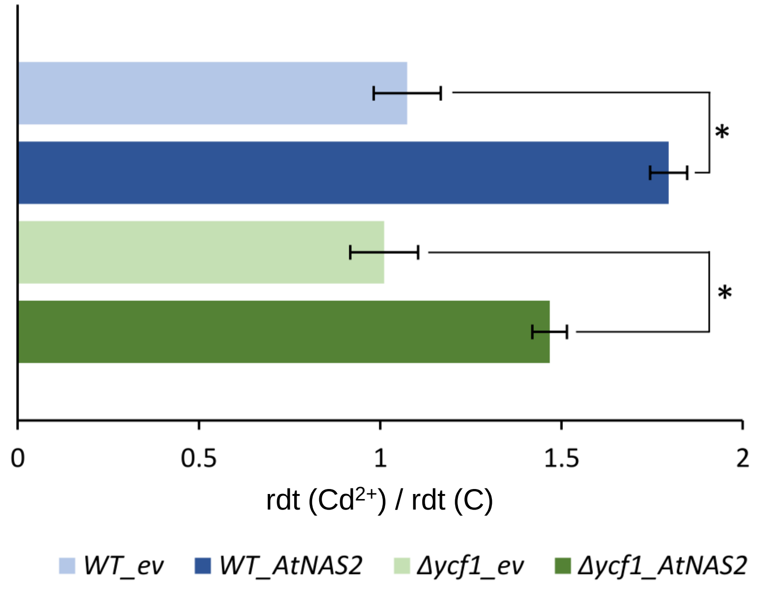

Suppl. Fig. S1: NA synthesis causes Cd hypersensitivity in *S. cerevisiae*. AtNAS2 was expressed in WT and Cd hypersensitive *Δycf1* cells. Growth was compared to cells carrying empty vector (ev). Doubling time of cells cultivated in the presence of either 5 µM Cd^{2+} (wild type, WT) or 1 µM Cd^{2+} (*Δycf1*) relative to cells cultivated under control conditions (rdt = relative doubling time). Shown are mean values +/- SD (n = 3 independent experiments). Data were analyzed by t-test, * $p < 0.05$.

Suppl. Fig. S2

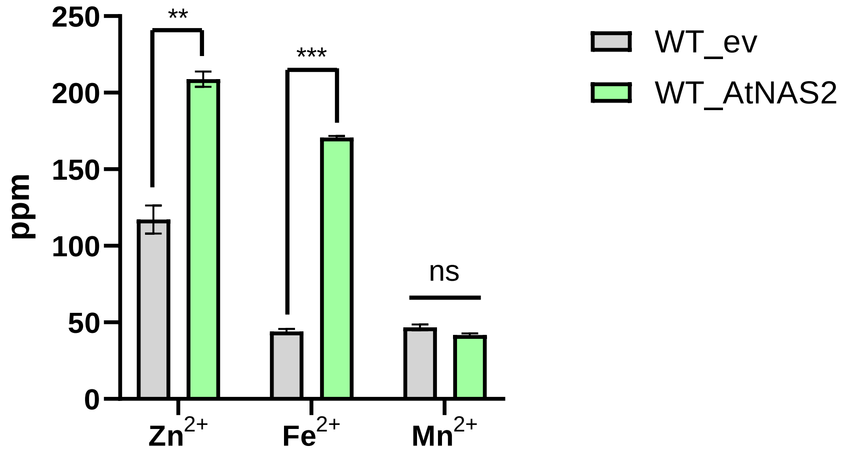

Suppl. Fig. S2: NA synthesis enhances Zn and Fe accumulation in *S. pombe*. Wild-type cells carrying the empty vector (ev) or expressing AtNAS2 were grown under standard conditions. Zn, Fe, and Mn content of cells was determined after 17 h. Shown are means +/- SD (n = 3 independent experiments). Data were analyzed by t-test, ** $p < 0.01$; *** $p < 0.001$; n.s. = not significant.

Suppl. Fig. S3

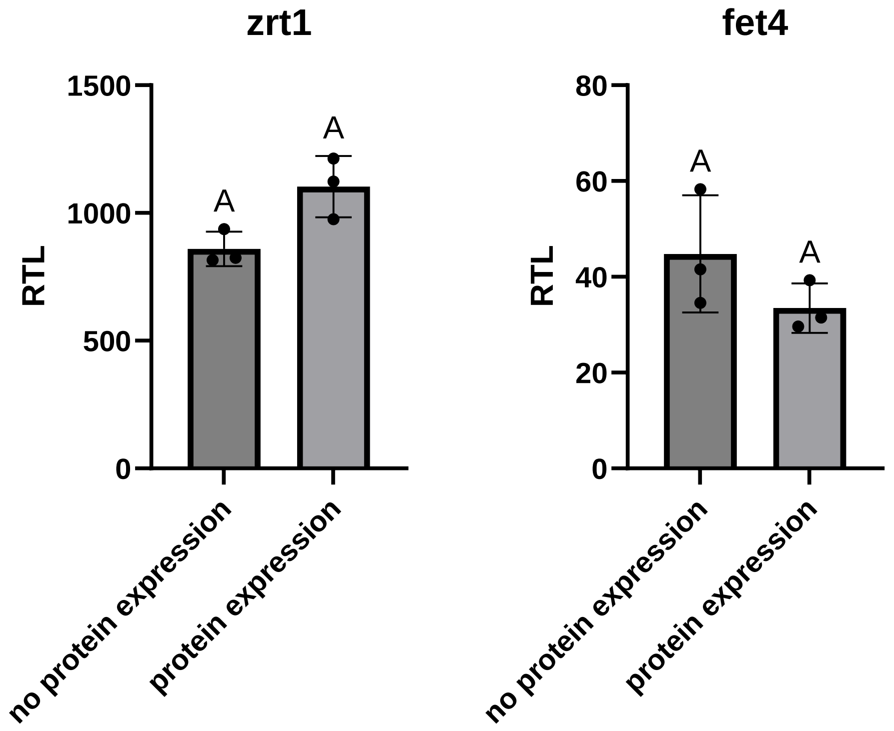

Suppl. Fig. S3: Transcript abundance of the two *S. pombe* Zn deficiency marker genes *zrt1* and *fet4* is not affected by AtNAS2 expression. RT-qPCR analysis of *zrt1* and *fet4* transcript abundance in cells expressing AtNAS2 (no thiamin in the medium) or not expressing AtNAS2 (20 µM thiamine). Data were normalized to the *act1* gene (RTL = relative transcript level) and analyzed by one-way ANOVA and Tukey's post-hoc test.

Suppl. Fig. S4

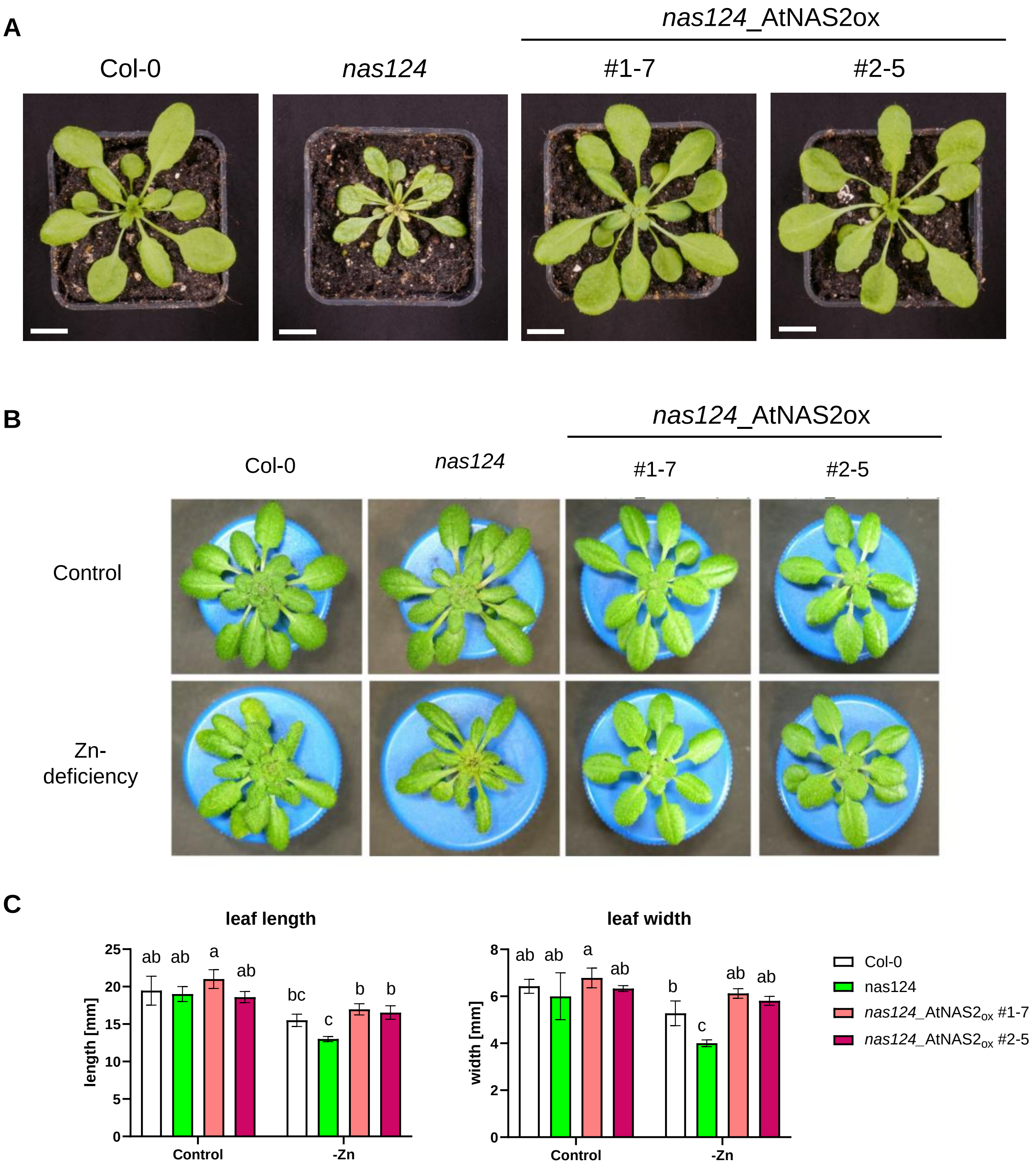

Suppl. Fig. S4: AtNAS2 overexpression rescues the *chloronerva* phenotype of the *nas1nas2nas4* triple mutant and confers wild-type growth under Zn deficiency conditions. A. Rosettes of Col-0, *nas1nas2nas4* triple mutant plants and two independent transgenic lines overexpressing AtNAS2 in triple mutant background. B. Growth of the four genotypes in hydroponic culture under Zn replete (control) and Zn deplete (Zn deficiency) conditions. C. Quantification of leaf growth under Zn replete and Zn deplete conditions; left: leaf length; right: leaf width. From each plant the 7 to 10 longest leaves were chosen. Shown are means +/- SD (n = 3 independent experiments). Data were analyzed by two-way ANOVA and Tukey's post-hoc test. Letters indicate significant differences ($p < 0.05$).

Suppl. Fig. S5

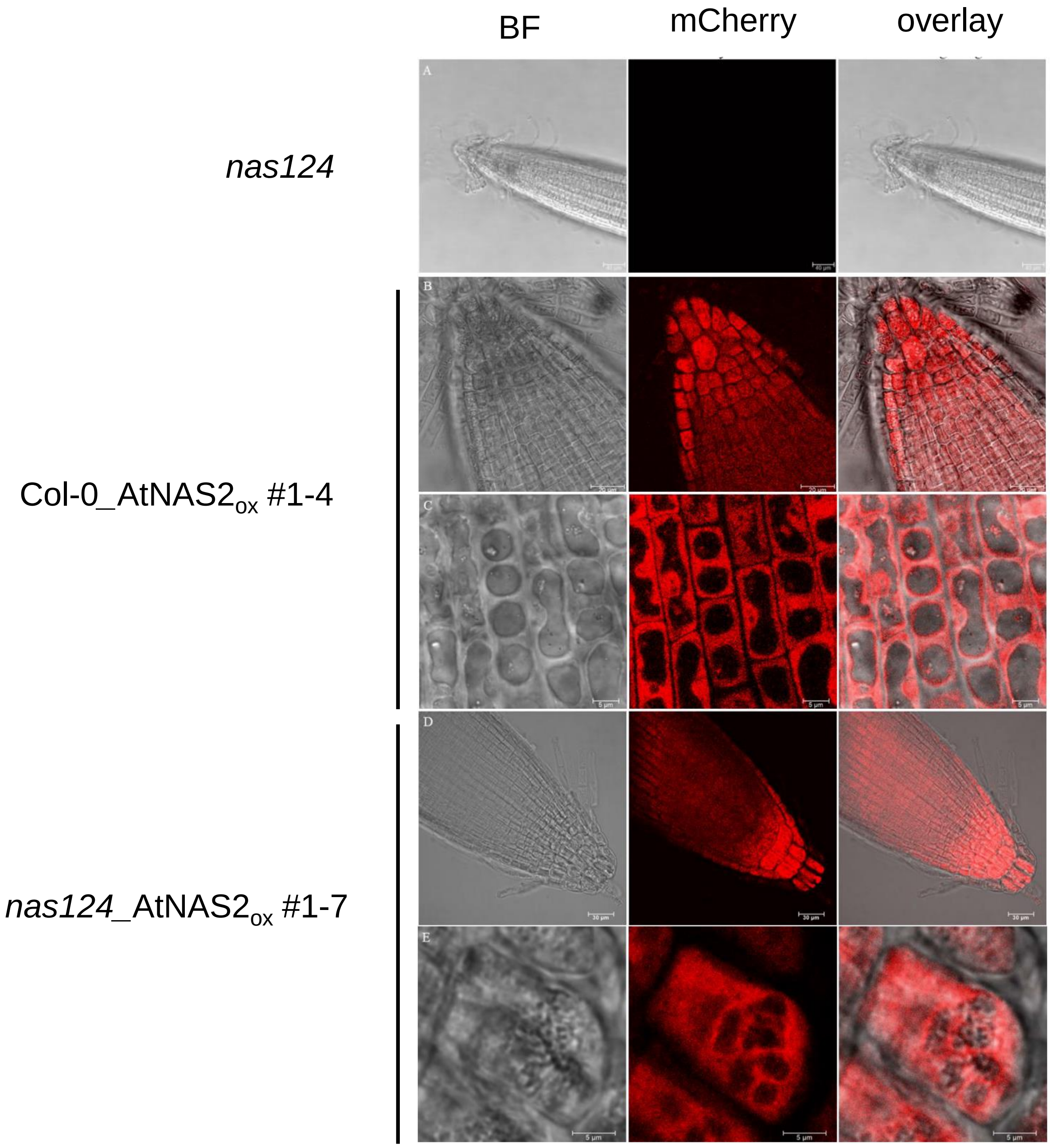

Suppl. Fig. S5: Cytosolic localization of mCherry-AtNAS2 expressed under control of the UBQ10 promoter. The mCherry fluorescence was detected in root tips of two representative transgenic lines in Col-0 and *nas1nas2nas4* triple mutant background by confocal microscopy. Left: brightfield image (BF); center: mCherry signal; right: overlay. In the top row an untransformed *nas1nas2nas4* triple mutant is shown. Scalebars are A: 40 µm; B: 20 µm; C and E: 5µm; D: 30µm.

Suppl. Fig. S6

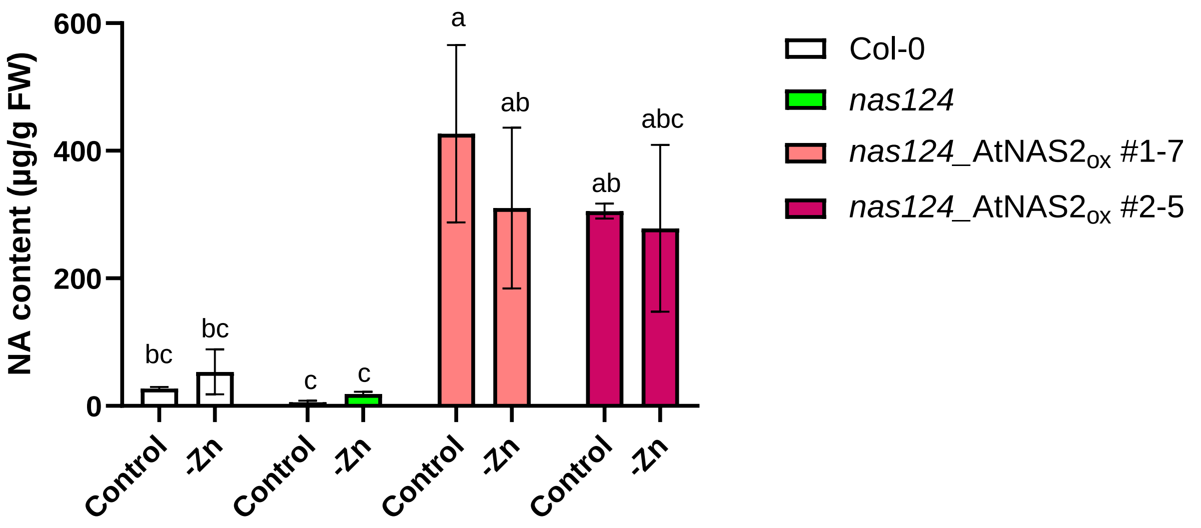

Suppl. Fig. S6: Leaf NA concentrations in Col-0, the *nas1nas2nas4* triple mutant and two overexpression lines. Plants were cultivated hydroponically under Zn replete (control) or Zn deplete (-Zn) conditions. NA concentrations in leaves were measured by HPLC after Fmoc derivatization. Shown are means +/- SD (n = 3 independent experiments). Data were analyzed by two-way ANOVA and Tukey's post-hoc test. Letters indicate significant differences ($p < 0.05$).

Suppl. Fig. S7

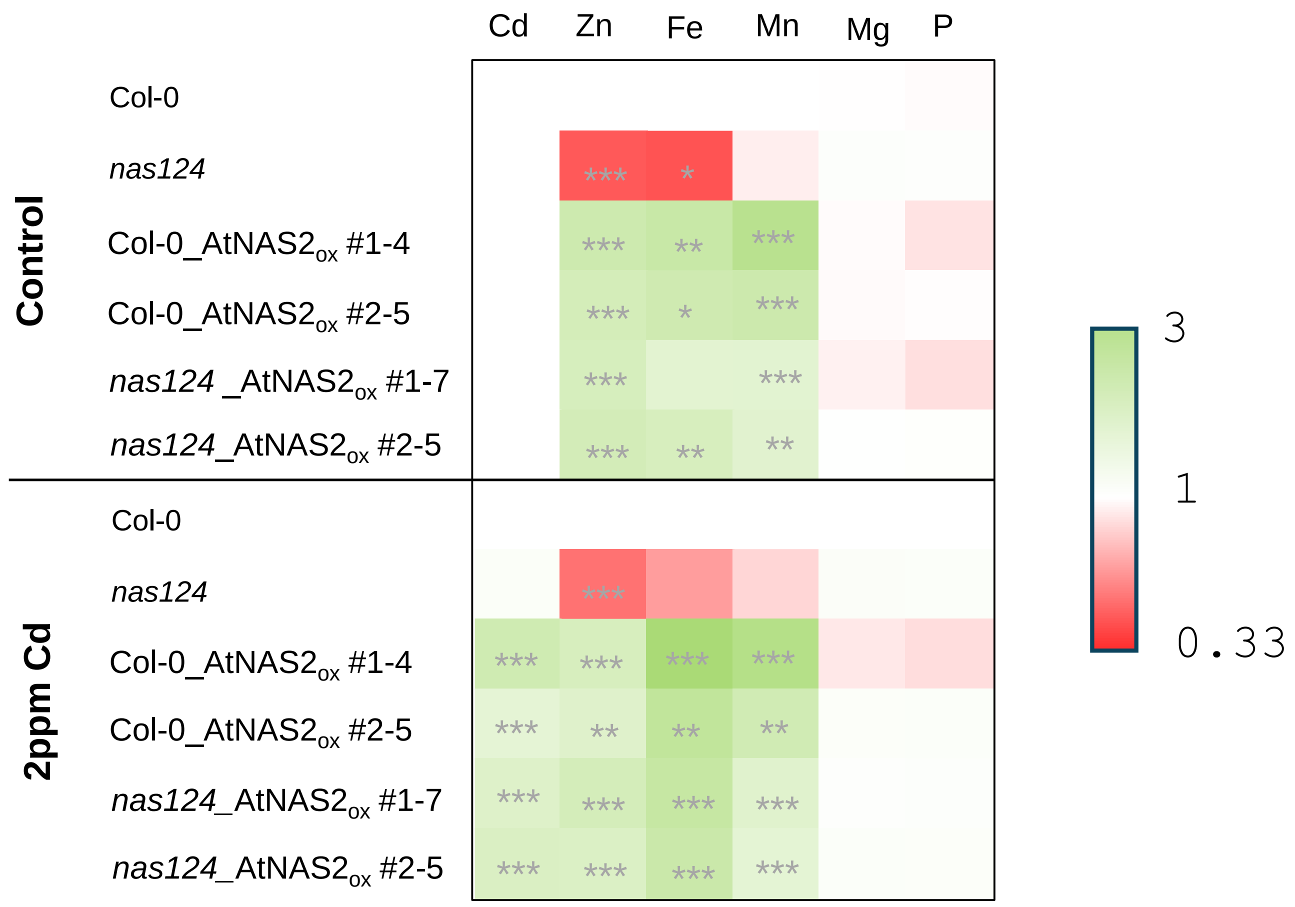

Suppl. Fig. S7: Effects of NA-overproduction on the accumulation of selected macro- and microelements as well as Cd in seeds. Col-0 and *nas1nas2nas4* plants (*nas124*) as well as two independent *NAS2* overexpression lines in the two genetic backgrounds were cultivated to maturity either on control soil or on soil artificially contaminated with Cd to an environmentally relevant extent (see Fig. 7). Ripened seeds were subjected to ICP-OES analysis. Data were normalized to Col-0 values. For each element and condition, the mean for Col-0 was set to 1. Shown are means +/- SD (10 – 15 individual plants cultivated in 3 independent experiments). Data were analyzed with the Kruskal-Wallis test followed by Bonferroni post-hoc correction; *** adj. p-value < 0.001, ** adj. p-value < 0.01, * adj. p-value < 0.05.

Suppl. Fig. S8

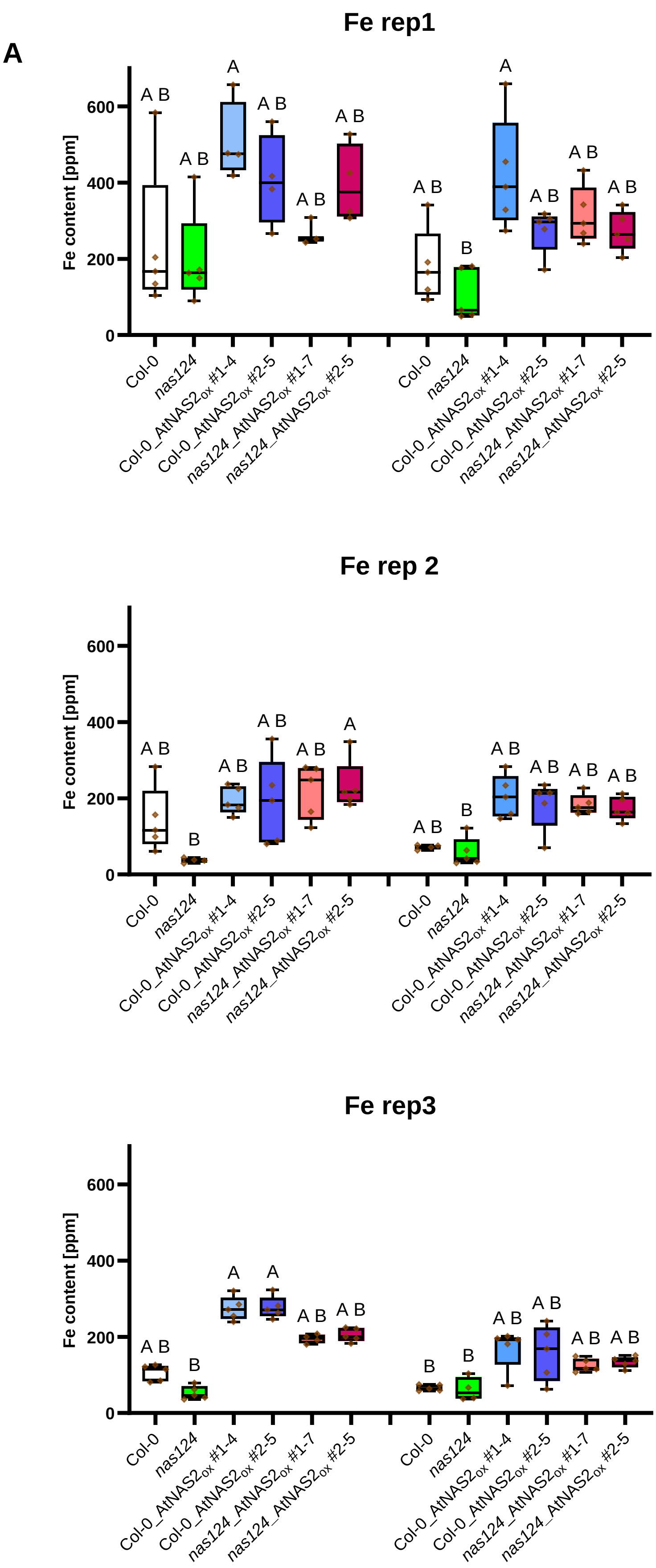
A
Fe rep1
Fe rep 2
Fe rep3
Fe content [ppm]
0
200
400
600
Col-0
nas124
Col-0_AtNAS2ox #1-4
Col-0_AtNAS2ox #2-5
nas124_AtNAS2ox #1-7
nas124_AtNAS2ox #2-5
A B
A
B

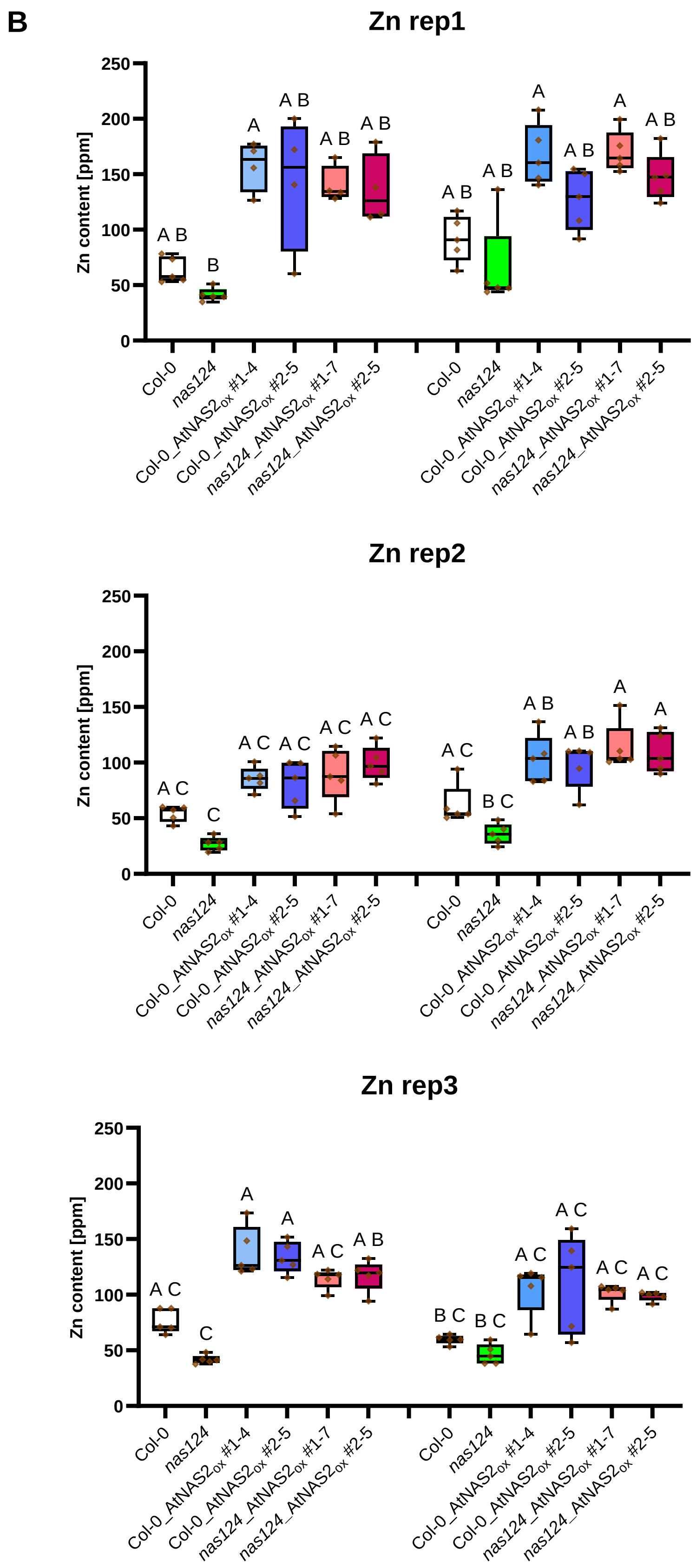
B
Zn rep1
Zn rep2
Zn rep3
Zn content [ppm]
Col-0
nas124
Col-0_AtNAS2ox #1-4
Col-0_AtNAS2ox #2-5
nas124_AtNAS2ox #1-7
nas124_AtNAS2ox #2-5

C

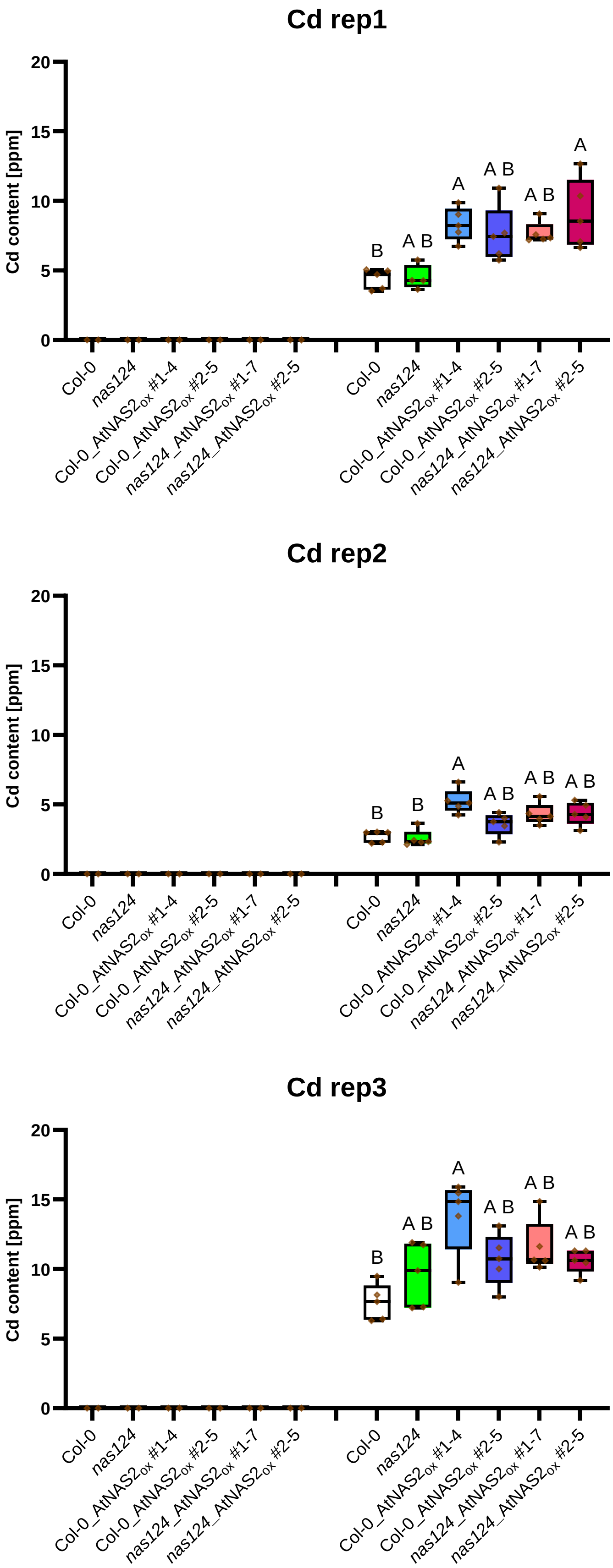
Cd rep1
Cd rep2
Cd rep3
Cd content [ppm]
0
5
10
15
20
Col-0
nas124
Col-0_AtNAS2ox #1-4
Col-0_AtNAS2ox #2-5
nas124_AtNAS2ox #1-7
nas124_AtNAS2ox #2-5
A
B
A B

Suppl. Fig. S8: Effects of NA-overproduction on the accumulation of Fe, Zn and Cd in seeds. Absolute values for the three independent experiments with Col-0 and *nas1nas2nas4* plants (*nas124*) as well as two independent NAS2 overexpression lines in the two genetic backgrounds cultivated to maturity either on control soil or on soil artificially contaminated with Cd to an environmentally relevant extent (A: Fe; B: Zn; C: Cd). Shown are means +/- SD (3 – 5 individual plants per replicate). Data were analyzed with the Kruskal-Wallis test followed by Bonferroni post-hoc correction. Letters indicate significant differences ($p < 0.05$).
